# Human IgG is produced in a pro-form that requires clipping of C-terminal lysines for maximal complement activation

**DOI:** 10.1080/19420862.2015.1046665

**Published:** 2015-06-02

**Authors:** Ewald TJ van den Bremer, Frank J Beurskens, Marleen Voorhorst, Patrick J Engelberts, Rob N de Jong, Burt G van der Boom, Erika M Cook, Margaret A Lindorfer, Ronald P Taylor, Patrick HC van Berkel, Paul WHI Parren

**Affiliations:** 1 Genmab; Utrecht, The Netherlands; 2 Department of Biochemistry and Molecular Genetics; University of Virginia School of Medicine; Charlottesville, VA, USA; 3 Department of Cancer and Inflammation Research; Institute of Molecular Medicine; University of Southern Denmark; Odense, Denmark; 4 Department of Hematology and Blood Transfusion; Leiden University Medical Center; Leiden, The Netherlands

**Keywords:** complement activation, herapeutic antibody, post-translational control

## Abstract

Human IgG is produced with C-terminal lysines that are cleaved off in circulation. The function of this modification was unknown and generally thought not to affect antibody function. We recently reported that efficient C1q binding and complement-dependent cytotoxicity (CDC) requires IgG hexamerization at the cell surface. Here we demonstrate that C-terminal lysines may interfere with this process, leading to suboptimal C1q binding and CDC of cells opsonized with C-terminal lysine-containing IgG. After we removed these lysines with a carboxypeptidase, maximal complement activation was observed. Interestingly, IgG1 mutants containing either a negative C-terminal charge or multiple positive charges lost CDC almost completely; however, CDC was fully restored by mixing C-terminal mutants of opposite charge. Our data indicate a novel post-translational control mechanism of human IgG: human IgG molecules are produced in a pro-form in which charged C-termini interfere with IgG hexamer formation, C1q binding and CDC. To allow maximal complement activation, C-terminal lysine processing is required to release the antibody's full cytotoxic potential.

## Abbreviations

ADCC: antibody-dependent cell-mediated cytotoxicity
CDC: complement-dependent cytotoxicity
CEX: cation-exchange
cIEF: capillary isoelectric focusing
CPB: carboxy peptidase B
ESI-MS: electrospray ionization mass spectrometry
IEF: isoelectric focusing
SDS-PAGE: sodium dodecyl sulfate polyacrylamide gel electrophoresis

## Introduction

Therapeutic antibodies have emerged as important drugs for the treatment of cancer, autoimmune disease, infection and cardiometabolic disorders,^1-4^ and they represent one of the fastest growing product segments of the pharmaceutical industry. The safety and efficacy of therapeutic monoclonal antibodies (mAbs) is therefore extensively studied, with a strong focus on the various post-translational modifications that cause product heterogeneity.^5–7^ One common modification is the processing of the heavy chain C-terminal lysine, which was first described more than 20 years ago.^8,9^ Minimizing variations in C-terminal lysine processing can facilitate monitoring of batch-to-batch product variation. Interestingly, the C-terminal lysine is conserved in the heavy chain genes of all human IgG subclasses (i.e., IgG1, IgG2, IgG3 and IgG4), yet this residue is generally absent from IgG found in serum.^10,11^ Recently, the fate of the C-terminal lysine of a recombinant human IgG2 was studied in vivo and found to be rapidly lost with a half-life of approximately one hour.^12^ Because of the distal location of the heavy chain C-terminus, away from the antigen-binding domain^13^ as well as the sites involved in interactions with effector molecules such as Fcγ receptors^14,15^ and complement C1q,^8,14,16^ it is generally thought that C-terminal lysines do not affect antibody function. In support of this notion, no substantial conformational changes between C-terminal lysine variants were observed with hydrogen-deuterium exchange mass spectrometry.^17^ A study that compared several batches of a humanized Lewis-Y IgG1 containing distinct C-terminal lysine variants, albeit at relatively low concentration, concluded that C-terminal lysines did not affect CDC.^18^ Nevertheless, the few available studies may have missed the effect of C-terminal processing on antibody function because they were performed with mixtures rather than purified antibody fractions.

Here, we report our investigation of whether the C-terminal lysines of human IgG affect antibody-induced complement activation. We purified isoforms of CD20 and CD38 mAbs carrying zero, one or 2 heavy chain C-terminal lysines per antibody molecule (further designated as K0, K1 and K2 isoforms) and compared their ability to induce CDC. Clearly, the K0 isoform of both antibodies showed significantly increased CDC activity compared to the K2 variant. This conclusion was confirmed by studying CDC with engineered antibodies containing no charge, a negatively charged (site chain) amino acid, or 1–3 positively charged (site chain) amino acids at their C-termini. CDC activity was lost as mutants carried more C-terminal positive charges. Interestingly, also addition of the negative charges decreased CDC, which could be fully restored in antibody mixtures of C-terminal mutants with opposite charge. Restoration of CDC activity was dependent on the stoichiometry of the mutants in the mixture. Taken together, our work shows that human IgGs are produced as a pro-form and that removal of C-terminal lysine from the heavy chain is required for mediating maximal CDC. The results are consistent with our observation that antibodies require the formation of ordered hexameric structures for optimal complement activation potency.

## Results

### Isolation of hybridoma-derived human IgG1 C-terminal lysine isoforms

**Figure 1.**
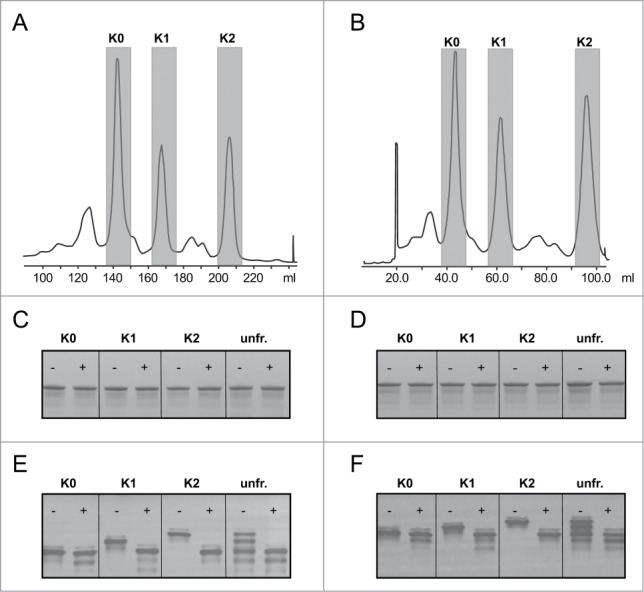
CEX fractionation profiles of hybridoma-derived batches of a CD20 mAb (**A**) and a CD38 mAb (**B**). Fractions containing K0, K1 and K2 isoforms were collected and pooled. Fractions are highlighted in gray. The fractions were incubated with (+) or without (−) carboxypeptidase B (CPB) to demonstrate the presence of C-terminal lysines and analyzed the samples on SDS-PAGE (**C, D**) and IEF gel electrophoresis (**E, F**). The changes in charge on IEF gel suggest the absence of C-terminal lysines in K0, and the presence of one lysine in K1 and 2 lysines in K2. Unfr. indicates the original unfractionated material.

Human mAbs obtained from hybridoma cell lines contain significant amounts of charged isoforms when analyzed by cation exchange chromatography (CEX), which may represent C-terminal lysine heterogeneity as previously shown by Dick et al.^19^ CEX is the general standard used to study mAb charge variants.^20^ Therefore, isoforms of a CD20 (HuMAb 2F2^21^) and a CD38 (HuMAb 005^22^) mAb were isolated with preparative CEX and main peaks were collected (**Fig. 1A, B**). Biochemical analysis by SDS-PAGE (**Fig. 1C, D**) and isoelectric focusing (IEF) (**Fig. 1E, F**) indicated that the structural integrity was as expected, and that the different main peaks represented IgG charge isoforms of the mAbs. ESI-MS analysis of the glycosylated heavy chain showed that the glycan profiles were similar between the different isoforms (**Fig. S1A–D**). To confirm that the charge variation was due to C-terminal lysine heterogeneity, aliquots were treated with carboxypeptidase B (CPB), which specifically cleaves C-terminal lysines.^19^ The IEF analysis of enzyme-treated and untreated samples (**Fig. 1E, F**) clearly indicated that the observed charge heterogeneity resulted from C-terminal lysines as CPB treatment of the K1 and K2 isoforms of both antibodies generated the K0 isoform. The relative composition of the C-terminal isoforms (as determined by CEX) was 29%, 21% and 26% of K0, K1 and K2 isoforms for the CD38 mAb, and 33%, 19% and 18% for the CD20 mAb, respectively (**Fig. 1A, B**). The remaining 24% and 30% of the CD38 and CD20 mAb, respectively, likely representing acidic isoforms, were not further characterized nor analyzed in this study.

### CDC of isolated C-terminal lysine isoforms

**Figure 2.**
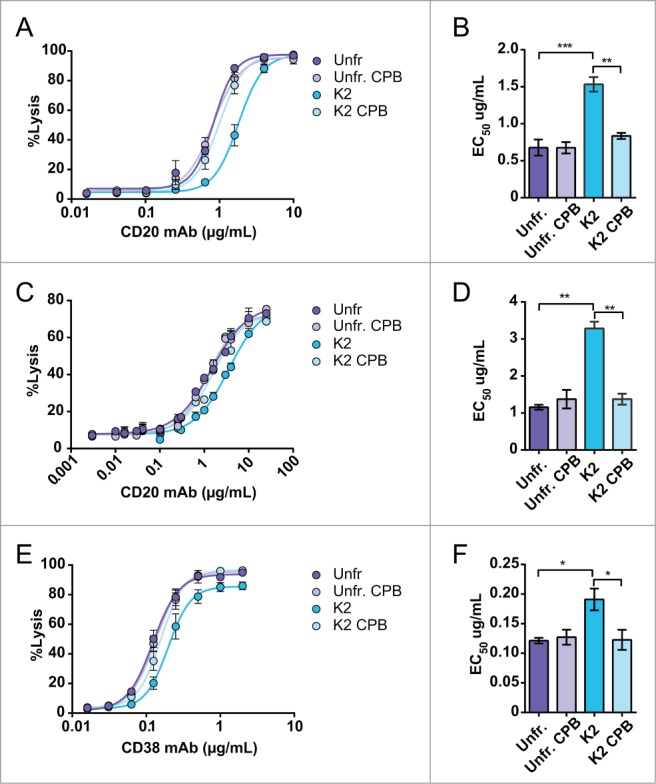
Dose-response curves and EC50 values of complement-mediated lysis (CDC) induced by the CD20 mAb of Daudi cells (**A, B**; n = 3) and of Raji cells (**C, D**; n = 2) and induced by the CD38 mAb of Daudi cells (**E, F**; n = 3). For both mAbs the unfractionated (purple symbols and bars) and K2 isoform (blue symbols and bars) with and without CPB treatment were analyzed. NHS (20% vol/vol) was used as complement source and cells were incubated at 37°C for 45 min. Lysis was assessed by flow cytometry using a PI exclusion assay and the level of CDC is expressed as percentage of total cells. The data represents mean ± SEM and statistical significance was assessed by one-way ANOVA followed by a Tukey post hoc test (*p < 0.05, **p < 0.01, ***p < 0.001).

To investigate the effect of C-terminal lysine clipping on cytotoxic activity, we compared the CDC activity of the unfractionated CD20 and CD38 mAbs and isolated isoforms on Daudi and Raji cells. Both mAb 2F2 and 005 have previously been shown to strongly induce complement activation and CDC.^21,22^ The dose-response curves and EC50 values were determined for both the CD20 and CD38 mAbs (**Fig. 2**). The CDC activity of the K0 and K1 isoforms were comparable to the unfractionated material (data not shown). However, the K2 isoforms of both mAbs showed an approximate 2–3 fold increase in EC50 value for CDC of Daudi or Raji cells compared to unfractionated mAb (**Fig. 2A–F**). CPB treatment of the K2 isoforms released the full ability to induce complement lysis with EC50 values similar to those obtained for unfractionated antibody. Notably, no significant changes were observed in CDC activity before and after CPB treatment of the unfractionated material for both CD20 and CD38 mAbs despite the large amount of C-terminal lysine K2 isoform present, i.e., 26% and 18%, respectively. This is also consistent with the observation that the purified K0 isoform performed similarly in CDC compared to the unfractionated material (data not shown).

**Figure 3.**
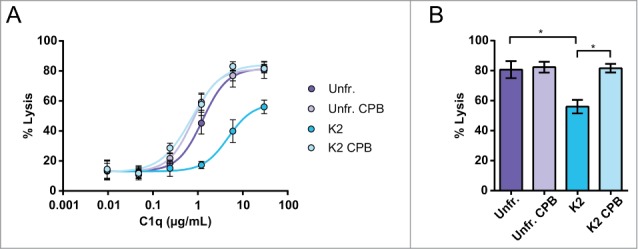
C-terminal lysine mediated inhibition of CDC results from less effective use of C1 when C1q was titrated in C1q depleted serum. (**A, B**; n = 3). The unfractionated (purple symbols and bars) and K2 isoform (blue symbols and bars) with and without CPB treatment were analyzed. Daudi cells were incubated at 37°C for 45 min and lysis was assessed by flow cytometry using a PtdIns exclusion assay. The level of CDC is expressed as percentage of total cells. The absence of CDC without added C1q indicates classical pathway activation. The data represents mean ± SEM and statistical significance was assessed for maximal lysis by one-way ANOVA followed by a Tukey post hoc test (*p < 0.05).

To exclude the possibility that altered binding properties explained the differences in CDC capability, flow cytometric binding analysis of the isolated isoforms to Daudi and Raji cells were performed. The results demonstrated that antigen binding was comparable between unfractionated antibody and all isoforms (binding K0 and K1 not shown), either before or after CPB treatment (**Fig. S2A–C**). To further investigate the basis of the reduced CDC activity in C-terminal lysine-containing mAbs, we examined activation of the classical complement pathway by the CD38 mAb in more detail. Hence, CDC experiments were performed with C1q-depleted serum in which C1q was titrated (**Fig. 3A, B**). The absence of CDC without added C1q indicates classical pathway activation. Clearly, the K2 isoform of the CD38 mAb required substantially higher concentrations of C1q and did not reach the same maximal lysis (56%) as unfractionated mAb (81%) unless it was C-terminally cleaved by CPB (82%) (*p* < 0.05).

### CDC of C-terminal heavy chain mutants

To further strengthen our observations, 5 C-terminal heavy chain mutants of the CD38 mAb were constructed (**Table S1**) and expressed in HEK-293F cells next to wild-type CD38 mAb 005.^22^ First, a C-terminal mutant was constructed lacking C-terminal charges, and therefore mimicking the K0 isoform, i.e., ending with the sequence -PG. Furthermore, 3 lysine-containing mutants were prepared with one, 2 and 3 consecutive lysines at the C-terminal end of the heavy chains. To prevent C-terminal lysine clipping during production, the final C-terminal lysine was capped by a proline, providing the sequences -PGKP (K2); -PGKKP (K4) and -PGKKKP (K6). To further examine the effect of charge, one mutant, -PGE (E2), was produced with a negatively charged glutamic acid instead of positively charged lysine. Glutamic acid is not sensitive to carboxypeptidase activity, and therefore no proline was added.

**Figure 4.**
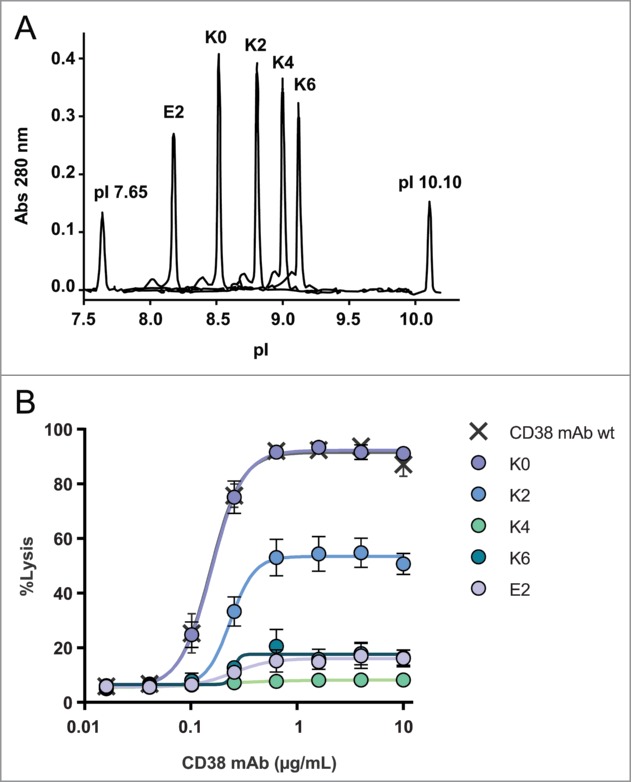
Overlay of capillary isoelectric focusing profiles of CD38 C-terminal mutants (**A**). The mutant abbreviations K0, K2, K4, K6 and E2 (**Table S1**) and pI markers 7.65 and 10.10 are indicated and detection occurred at 280 nm. Dose-response curves of CDC mediated cell lysis of the C-terminal mutants (n = 3) (**B**). NHS (20% vol/vol) was used as complement source and cells were incubated at 37°C for 45 min. Lysis was assessed by flow cytometry using a PI exclusion assay and the level of CDC is expressed as percentage of total cells. CD38 mAb wt produced in HEK-293F was used as positive control.

To check whether the mutants had different overall charges, capillary isoelectric focusing profiles were obtained (**Fig. 4A**) and the pI values are summarized (**Table S1**). The pI shifted from 8.2 for E2 to 9.1 for K6, in line with the expected pI based on the amino acids introduced. Furthermore, SDS-PAGE and HP-SEC results showed that the structural integrity was maintained and the N-linked glycosylation was similar for all C-terminal mutants (data not shown). In addition, all mutants bound equally to Daudi cells, and were comparable to the binding of wild-type CD38 mAb produced in HEK-293F (**Fig. S3**).

We next addressed the ability of these mutants to induce CDC (**Fig. 4B**). The CDC potency of the wild-type CD38 mAb, which is produced in HEK-293 cells and, in contrast to hybridoma-derived material, is predominantly clipped, and the K0 mutant were virtually identical, with a maximal lysis (top value) of ∼95% specific lysis, whereas the K2 mutant showed significantly reduced CDC with a maximal lysis of ∼50%. Surprisingly, the K4 mutant completely lost CDC activity, whereas the K6 variant showed strongly reduced CDC potency with maximal lysis of ∼15%. The negatively charged mutant E2 also showed strongly reduced CDC potency, with a top value of ∼15%.

**Figure 5.**
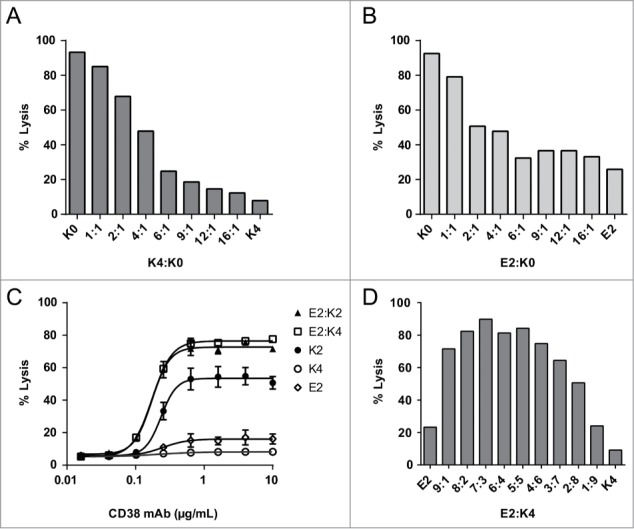
Maximal CDC obtained with the C-terminal K0 and K4 mutant and combinations thereof (**A**) and for K0 in combination with E2 (**B**). Dose-response curves of complement-mediated lysis for E2:K2 and E2:K4 mixed in a 1:1 ratio (n = 2) and individual mutants (n = 3). The data represents mean ± SEM (**C**). Maximal CDC obtained with mixtures of E2 and K4 mutants at a total IgG concentration of 10 μg/mL (**D**). NHS (20% v/v) was used as complement source and cells were incubated at 37°C for 45 min. Lysis was assessed by flow cytometry using a PtdIns exclusion assay and the level of CDC is expressed as percentage of total cells.

We recently showed that C1 binding and subsequent complement activation occurs most optimally when IgG molecules assemble into ordered hexameric structures at the cell surface.^23^ We therefore reasoned that charge repulsion between the C-terminal lysine residues of neighboring IgG molecules might be the basis of our observations. To explore this, we performed CDC experiments using mixtures of isoforms containing opposite C-terminal charges. First, we tested the effect of the K4 and E2 mutants on the CDC activity when mixed with K0 (**Fig. 5A, B**). Interestingly, CDC decreased when the K4:K0 or E2:K0 ratios were increased while keeping the total IgG concentration constant. Remarkably, CDC activity only decreased significantly when the K4:K0 or E2:K0 ratios increased above 1 with a maximum at about a 6 to 16-fold excess of the charged over the non-charged isoform for the E2 and K4 variants, respectively. This observation may explain why a decrease in CDC was not observed for the unfractionated CD20 and CD38 mAb, which only contained 26% and 18% of the charged K2 isoform, respectively.

If repulsion between C-terminal charges on IgG molecules affects C1q binding and CDC, then mixing molecules with oppositely charged C-termini might neutralize that effect. Indeed, mixing the negatively charged E2 isoform with the positively charged K2 isoform in equimolar amounts almost fully restored CDC to top values of about 80% (**Fig. 5C**). A similar result was obtained when the very poorly complement-activating K4 mutant was mixed with E2 (i.e., E2:K4), recovering CDC activity to top values comparable to those obtained for 1:1 combinations of K4:K0 and E2:K0 (**Fig. 5A, B**). To demonstrate that CDC occurred via classical pathway activation, we analyzed C1q binding and C3 deposition on Daudi cells opsonized with an equimolar mixture of K4 and E2 or the K0 isoform alone. These experiments confirmed that mixing the complement-deactivating mutants K4 and E2 largely restored C1q binding and C3b deposition to the levels observed for K0 (**Fig. S4**).

A titration experiment was conducted by testing different ratios of E2 and K4. Maximal lysis occurred at equimolar stoichiometry with a trend of an optimum requiring somewhat more E2 compared to K4 (**Fig. 5D**). Finally, because CD20 and CD38 are highly expressed on Daudi and Raji cells, we investigated non-specific complement activation for the K0 and K2 isoforms of CD38. The results show that there are no differences between K0 and K2 when C1q binding and C3b deposition are measured after randomized coating in an ELISA format followed by addition of varying dilutions of normal human serum (NHS) (**Fig. S5 and S6**). Similarly, there was no difference in complement consumption in fluid phase (CH50 assays) after the K0 and K2 forms were modestly aggregated by heating (not shown).

## Discussion

Here, we describe for the first time that complement activation and CDC induced by human IgG is increased >2–3 fold, when C-terminal lysines are cleaved from both heavy chains via post-translational modification. This was observed by comparing purified K0 and K2 isoforms of antibodies targeting human CD20 as well as CD38, indicating that the observed reduction in CDC potency was not target specific (**Figs. 2 and 3**). In addition, antibody preparations containing K2 isoforms gained CDC activity, comparable to that of the K0 isoform, upon removal of the C-terminal lysines by CPB treatment. Human IgG isoforms carrying one C-terminal lysine (K1) had a CDC response similar to that obtained for the completely clipped K0 isoform. Also, unfractionated hybridoma-derived batches containing significant amounts of both K1 and K2 isoforms showed CDC activity comparable to the K0 isoform. These data confirm previous results obtained for antibodies against the Lewis-Y antigen for unfractionated IgG batches before and after CPB treatment,^18^ and explain why the inhibitory activity of C-terminal lysines was not previously noticed. Likewise, Yang et al. tested IgG1 charge variants for CDC, and, although a slight decrease for the K2 variant was observed, this was explained by a slight difference in G1F glycan content.^24^ However, the CEX profile was poorly resolved for the K2 variant, which may explain why an effect of the C-terminal lysine remained unnoticed. The dependency on C1q when titrated into C1q-depleted serum indicates that the presence of C-terminal lysines negatively affects the classical pathway of complement activation (**Fig. 3**). Hence, C-terminal lysine clipping appears essential for antibodies to achieve their maximal complement-activating capability. In our IEF analysis (**Fig. 1E, F**), we noted the generation of minor acidic species by CPB treatment. This might be due to deamidation at elevated temperatures as treatment occurs at 37°C for 4 hours.^25^ Whether the acidic species represent deamidated isoforms is unclear. However, as the levels are low, an effect on CDC is unlikely.

Several C-terminal CD38 mAb mutants were generated to analyze the decreased CDC activity of the K2 isoforms in more detail. The K0 and K2 mutants confirmed our findings with fractionated hybridoma-derived material. The C-terminal addition of one or 2 additional positively charged lysines on each heavy chain (K4 and K6) further reduced CDC activity, likely due to increased electrostatic repulsion destabilizing either Fc-C1q or Fc-Fc interactions. However, a mutant with a negatively charged glutamic acid C-terminus (E2) also showed strongly reduced CDC, arguing against Fc-C1q repulsion. Interestingly, combining 2 antibody mutants with complementary C-terminal charges (e.g., E2 and K4), each with inferior CDC activity, largely restored CDC activity, as well as C1q binding and C3b deposition (**Figs. 4 and 5**). These observations show that activation of the classical pathway of complement is hampered by the electrostatic repulsion between similarly charged antibody C-termini.

**Figure 6.**
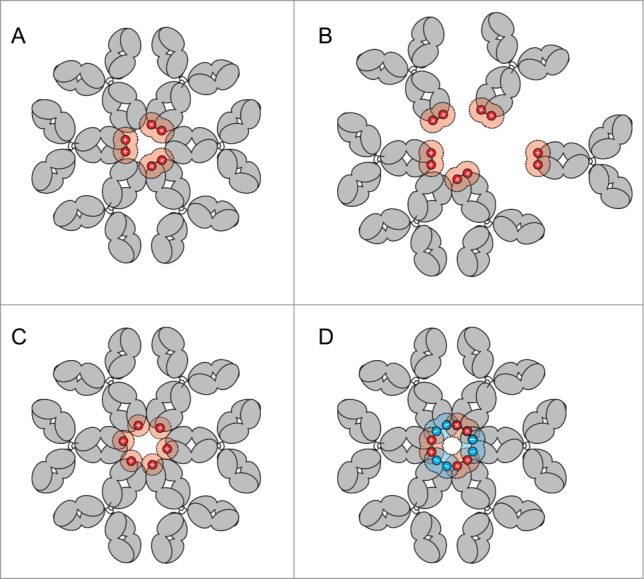
(**A**) Formation of IgG hexameric rings for effective complement activation at ˜50% K2. (**B**) Increasing K2 isoforms (>50%) bound to antigen, are incompatible with ordered hexamer formation because of charge repulsion between IgG molecules. (**C**) 100% K1 isoforms may form stable hexameric ring structures and do not show a decreased CDC activity. (**D**) Combining 2 antibody mutants with complementary C-terminal charges (e.g., containing C-terminal K and E), each with inferior CDC activity, allow the assembly into hexameric rings and thereby restore CDC activity.

Complement activation by the classical pathway is triggered by interaction of antibodies with C1q. This molecule has the appearance of a ‘bunch of tulips’ and is multivalent in its binding to IgG. This multivalency is probably the key to why complement is only triggered by IgG in an oligomeric form. Binding of C1q head pieces to a single IgG molecule is weak (K_d_ ∼10^−4^ M), whereas binding to oligomeric IgG and the consequent increase in avidity (K_d_ ∼10^−8^ M) allows physiological binding and activation of the complement cascade.^23,26^ Recently, we showed that Fc-Fc interactions between neighboring antibody molecules are essential for CDC activity and for formation of a hexameric recognition platform for C1.^23^ Hence, it is appealing to speculate that the formation of the hexameric ring is disturbed by charge-charge repulsion when both heavy chains contain a C-terminal lysine residue. Our results showed that at levels above ∼50% K2, CDC is decreased. This high percentage may be explained by the fact that IgG K2 molecules can assemble in alternating positions with K0, avoiding direct charge-charge repulsion when adopting a hexameric ring structure (**Fig. 6A**). Under conditions in which antibody molecules containing 2 C-terminal charges (K2) are in excess, assemblies that avoid charge clashes between neighboring molecules are no longer possible and hexamer formation may therefore be interrupted (**Fig. 6B**). The absence of a decrease in CDC for the K1 variant suggests that arrangements with alternating single charges are compatible with efficient hexamer formation and CDC (**Fig. 6C**), possibly because charged C-termini could point upwards or downwards relative to the hexameric Fc-plane in alternating fashion. Finally, the observation that combinations of negatively charged and positively charged C-termini completely restored complement activity is in agreement with our model of Fc-Fc cooperativity and hexamer formation (**Fig. 6D**). In support of this, Diebolder et al. showed that heavy chain charge variants in the vicinity of the C-terminus (i.e., K439E or S440K) resulted in a loss of C1q binding, while CDC could also be restored by mixing of these antibodies.^23^

Our data highlight that C-terminal lysine clipping may not simply be a redundant process, but part of a regulatory mechanism in which the K2 isoform of IgG is synthesized in a form in which complement activation capability is not yet fully activated. Upon release into the blood stream, IgG in humans is exposed to plasma carboxypeptidase N (CPN), a freely circulating form of CPB, which rapidly removes C-terminal lysines with an in vivo half-life of about 62 min, as measured for C-terminal lysine-containing isoforms of IgG2.^12^ Similarly, we observed polyclonal human IgG purified from human serum to be completely devoid of C-terminal lysines as we could not detect any in the Fc fragment of RhoGAM®; (a polyclonal human IgG preparation against rhesus antigen) by mass spectrometry (data not shown). Extrapolating, one might thus conclude that human IgG is encoded and produced in a pro-form that only acquires its full ability to activate complement following secretion and exposure to appropriate enzymes. Our findings might also hold relevance for murine IgG subclasses, in which the C-terminal lysine is fully conserved. It is intriguing to speculate that diminished CDC represents an adaption that may protect IgG-producing B-cells and plasma cells against lysis, as they continuously exist in very high antibody concentrations that might lead to the formation of antibody multimers. Additionally, it may protect the organism from undesired inflammatory effects of complement activation.

Therapeutic antibodies are important drugs used for a wide variety of diseases.^3,4,27^ Since the discovery of the hybridoma technology by Köhler and Milstein, mAbs obtained from hybridoma cells have been widely used.^28^ These antibodies contain significant amounts of unclipped C-terminal lysine heavy chains,^19^ sometimes as much as >90% (data not shown). Hybridoma-derived antibody libraries are also frequently used in antibody discovery processes to search for clinical candidates with or without potent CDC activity. As such libraries may be very heterogeneous in C-terminal lysine content, the selection process may be hampered and the final candidate may have altered functional properties once expressed in stable cell lines, e.g., CHO, SP2/0 or NS0 cell lines, where more extensive C-terminal lysine clipping will occur. Antibodies expressed in SP2/0 or NS0 may still incorporate incomplete lysine clipping,^29,30^ but at levels that are unlikely to reduce CDC. Although our data suggest that limited C-terminal heterogeneity does not affect complement activation, it should be noted that changes in charge heterogeneity due to new cell line generation may nevertheless be relevant for biosimilar development.^31^

For a number of non-mammalian IgG production platforms such as yeast, Lemna (duck weed) and Physcomitrella (moss), the glycosylation machinery is humanized, for instance to maintain or enhance ADCC effector function.^32-35^ However, besides N-linked glycosylation, C-terminal lysine cleavage also needs to be considered, especially in expression systems that lack efficient basic carboxypeptidases activity. The CDC activity of such antibodies might be underestimated when the full gene sequence is expressed, with the risk of unpredicted in vivo CDC activity in a clinical setting. Our data shows that CDC is fully activated when at least ∼50% of the C-terminal lysines are clipped. Upon infusion, this occurs during a one hour incubation time (t ½ = 62 min) in vivo.^12^ To achieve superior batch-to-batch consistency, the C-terminal lysine content of antibody products could be controlled by recombinantly removing the lysine before cell line development, or possibly by modifying cell culture trace element concentrations.^36,37^

Taken together, our studies show that the C-terminal lysine-containing heavy chain of IgG represents a pro-form in which complement activation via the classical pathway and CDC capability is down regulated. The C-terminal lysine thus acts as a molecular switch that requires clipping to activate the antibodies' full cytotoxic potential. Manipulation of the C-terminal ‘switch’ may open up alternative ways to regulate CDC.

## Materials and Methods

### Production of CD20 and CD38 hybridoma-derived mAbs

Hybridomas producing human mAbs against human CD38 (HuMab 005) and CD20 (2F2) were developed at Genmab and have been described previously.^21,22^ Hybridomas were expanded in tissue culture (HyQ medium; Perbio Sciences) and purified as described below.

### Production of CD38 C-terminal heavy chain mutants

Human IgG was expressed transiently in HEK-293F cells (Invitrogen). The coding regions of the VH and VL sequence of the antibody were cloned into expression plasmids pCONG1f0.4 and pCONKappa0.4 (Lonza), containing the human γ1 (allotype f) and human kappa light chain constant regions, respectively. HEK-293F cells were cultured in FreeStyle™ 293 Expression Medium (Invitrogen). Plasmids were transfected into HEK-293F cells using 293fectin™ according to manufacturer's instructions (Invitrogen). Site-directed mutagenesis was used to construct the C-terminal heavy chain mutants 445-PG-446, 445-PGKP-448, 445-PGKKP-449, 445-PGKKKP-450 and 445-PGE-447 (EU numbering conventions are used throughout the manuscript). Subsequently, antibodies were purified by Protein A affinity chromatography (MabSelect SuRe, GE Healthcare), dialyzed overnight to phosphate-buffered saline (PBS) and filtered over 0.2 μm dead-end filters. Concentrations of purified C-terminal IgG1 variants were determined by absorbance at 280 nm. Purified proteins were analyzed by SDS-PAGE, mass spectrometry to confirm structural integrity and similar N-linked glycosylation profiles.

### Isolation of C-terminal lysine isoforms by cation exchange chromatography

Preparative CEX was performed on an AKTA Purifier system using a ProPac WCX-10, 9 mm × 250 mm (Dionex), preparative column. Mobile phases A and B were 10 mM sodium phosphate (pH 7.2) and 25 mM sodium chloride in 10 mM sodium phosphate (pH 7.0), respectively. The human antibodies were dialyzed overnight prior to injection against mobile phase A. For the CD20 mAb, a linear gradient from 0% to 12% B in 60 minutes was used. For the CD38 mAb, a linear gradient from 8% to 13% B in 25 minutes was used. The flow rate for both antibody separations was set at 4 mL/min. and detection was performed at 280 nm. For each antibody, 6 consecutive injections were performed and the individual K0, K1 and K2 fractions were collected, concentrated (Vivaspin; 10,000 MWCO; Sartorius) and buffer exchanged into PBS buffer.

### Carboxypeptidase B (CPB) treatment

Each antibody sample was diluted with 20 mM sodium phosphate pH 7.2 to a concentration of 450 μg/mL. This solution (500 μL) was mixed with 10 μL of 0.05 IU/μL CPB (Calbiochem) and incubated for 4 h at 37°C. Samples were stored at −80°C until further use.

### Isoelectric focusing

Antibody samples were diluted to 5 μg/mL and 20 μL was loaded onto an IEF FocusGel 6–11 24S (ETC), according to the manufacturer's instructions. After the run, the gels were fixed with 20% trifluoroacetic acid at 30°C for 45 min. Detection of the bands was performed using ammoniacal silver staining procedure according to the FocusGel supplier. All other reagents and devices used for IEF were obtained from GE Healthcare.

### Capillary isoelectric focusing

Antibody samples were desalted prior to analysis. Final concentrations in the assay were 0.3 mg/mL IgG. The pI marker at pI 7.65 and pI 10.10 were used. Focusing was performed for 7 minutes at 3000 V and the whole-capillary absorption image was captured using the iCE280 from Convergent Bioscience. The data were analyzed by the EZChrom software supplied with the instrument.

### SDS-PAGE

Antibodies were analyzed on SDS-PAGE (4–12% Bis-Tris; Invitrogen) under non-reducing or reducing conditions at neutral pH according to the manufacturer's instructions. The gels were stained with Coomassie (Invitrogen) and digitally imaged using the Optigo Imaging System (Isogen).

### Antigen binding assay

Binding of CD20 and CD38 mAbs to human CD20 and CD38 expressed on B cells was determined using Raji and Daudi 1.0 × 10^5^ cells. Incubation occurred at 4°C with increasing concentrations of antibody diluted in RPMI 1640 medium containing 0.1% BSA (Roche Diagnostics). After 30 minutes, cells were washed twice in FACS buffer (PBS; B.Braun), 0.1% BSA and 0.02% sodium azide (Sigma-Aldrich) and incubated with a Fluorescein isothiocyanate (FITC)-conjugated rabbit-anti-human IgG for 30 minutes at 4°C. After washing, cells were suspended in FACS buffer and analyzed using a FACSCalibur flow cytometer (Becton Dickinson). Dose-response curves expressing the mean fluorescence intensity (MFI) of the FITC-signal versus the mAb concentration were plotted.

### C1q binding and C3b deposition on B cells opsonized with CD38 mAb mutants

Daudi cells were opsonized with mAbs utilizing the approach reported previously.^38^ Briefly, C1q binding was assessed on Daudi cells. Cells were opsonised with mAbs. All mAb concentrations were 20 μg/mL during opsonization and 10 μg/mL during incubation in 50% NHS. For the E2:K4 combination the total mAb concentration was 20 μg/mL during opsonization and 10 μg/mL during incubation in 50% NHS. The samples were subsequently probed with FITC anti-C1q for 30 min at room temperature. C3b deposition was assessed on Daudi cells. Cells were opsonized with C-terminal mutants and incubated in 50% NHS supplemented with the mAb at 10 μg/mL for 2.5 min or 30 min at 37°C. The reaction was stopped by addition of excess cold BSA/PBS. The cells were subsequently probed with a cocktail of FITC mAb 7C12 (specific for C3b/iC3b) and Al647 mAb 1H8 (specific for C3b/iC3b/C3dg).

### CDC and C1q efficacy assay

Cells were resuspended in test medium to a concentration of 2 × 10^6^ viable cell/mL. Daudi cells were used as target cells in a mAb-induced CDC assay. The cell suspension (50 μL) was incubated with 50 μL CD20 mAb in a serial dilution for 15 minutes at room temperature. After the incubation period, NHS (M0008, Sanquin, Amsterdam, The Netherlands) or C1q-depleted serum (Quidel) supplemented with low concentrations C1q (Complement Technologies) was added as a source of complement (final concentration 20%) to the cell suspension in round-bottom microtiter plates (Nunc). The mixture was incubated for 45 min at 37°C after which the reaction was stopped by placing the samples on ice. Propidium iodide (PI; Sigma Aldrich) was added as a means to visualize the DNA content which becomes accessible upon permeabilization of the cell membrane. The amount of PtdIns-positive cells is directly correlated to the amount of dead cells, and measured using flow cytometry.

### Tests for non-specific complement activation

Binding of C1q and deposition of C3b fragments on mAb-coated 96-well flat-bottomed plates (Costar) was measured based on standard ELISA formats.^39^ The K0 and K2 isoforms, as well as human IgM (Sigma), and either ofatumumab (OFA; anti-CD20 mAb)^40^ or rituximab (RTX; anti-CD20 mAb),^41^ were used to coat plates overnight at 4°C in bicarbonate buffer at concentrations between 1 and 10 μg/mL. The plates were then washed 3 times with PBS/0.05% Tween, allowed to dry, and then reacted with either neat NHS (for C3b deposition) or with a 20-fold dilution of NHS (for C1q binding),^21^ for 30 min at 37°C. The plates were then washed 3 times and allowed to dry. Mab 1H8, which is specific for C3b/iC3b/C3d,^40^ was biotinylated using standard procedures and used to detect deposited C3 fragments in the ELISA measurements based on secondary development with Streptavidin-Peroxidase (1/1000; Sigma). Polyclonal rabbit anti-human C1q (1/1000; Dako) was used to detect C1q binding based on secondary development with Peroxidase-conjugated goat anti-rabbit IgG (1/1500; Jackson).

### Statistical analysis

Statistical analysis was performed by one-way ANOVA followed by a Tukey post hoc test using GraphPad Prism 5.01. Results were considered significant when *p* < 0.05.
