# Supplementary material for: Human IgG is produced in a pro-form that requires clipping of C-terminal lysines for maximal complement activation
Source: MAbs. 2015 Jun 2;7(4):672–80. doi: 10.1080/19420862.2015.1046665 (PMC4622059; doi:10.1080/19420862.2015.1046665)
Supplement: Supplemental_Material.zip [file kmab-07-04-1046665-s001.zip › Supplemental Material.docx]

**Supplementary information for**

**Human IgG is produced in a pro-form that requires clipping of C-terminal lysines for maximal complement activation**

**Ewald T.J. van den Bremer^a^, Frank J. Beurskens^a^, Marleen Voorhorst^a^, Patrick J. Engelberts^a^, Rob N. de Jong^a^, Burt G. van der Boom^a,1^, Erika M. Cook^b^, Margaret A. Lindorfer^b^, Ronald P. Taylor^b^, Patrick H. C. van Berkel^a,2^ and Paul W.H.I. Parren^a,c,d,3^**

^a^Genmab, Utrecht, the Netherlands

^b^Department of Biochemistry and Molecular Genetics, University of Virginia School of Medicine, Charlottesville, VA 22908, USA

^c^ Department of Cancer and Inflammation Research, Institute of Molecular Medicine, University of Southern Denmark, Odense, Denmark

^d^Department of Hematology and Blood Transfusion, Leiden University Medical Center, Leiden, the Netherlands

^1^Current address: Batavia Bioservices, Zernikedreef 9, 2333 CK Leiden, the Netherlands

^2^Current address: ADC Therapeutics, Chemin de la Pacottaz 1,1806 St-Legier, Switzerland

^3^Corresponding author:

Paul W.H.I. Parren, PhD; Genmab, Yalelaan 60, 3584 CM Utrecht, The Netherlands; Phone: +31 30 2123106; Fax: +31 30 2123110; E-mail: [p.parren@genmab.com](mailto:p.parren@genmab.com)


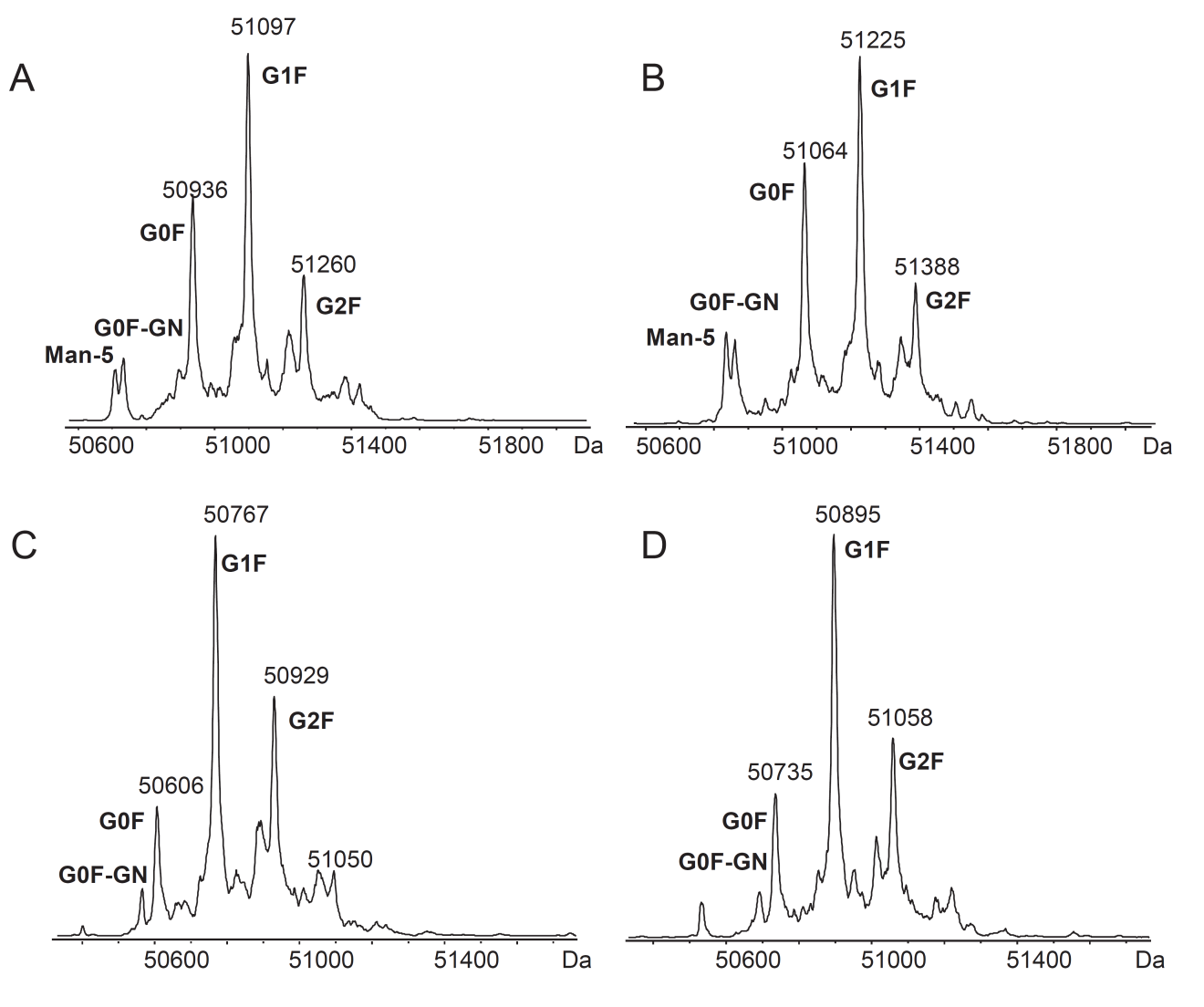


**Supplementary Figure 1.** ESI-MS analysis of the heavy chain N-linked glycan profile derived from the CD20 mAb isoforms K0 (A) and K2 (B). Spectra shown in (C) and (D) represent the heavy chain N-linked glycan profiles for the hybridoma derived CD38 mAb 005 isoforms K0 and K2, respectively. Main N-glycan peaks are indicated by G0-GN, G0F, G1F and G2F.


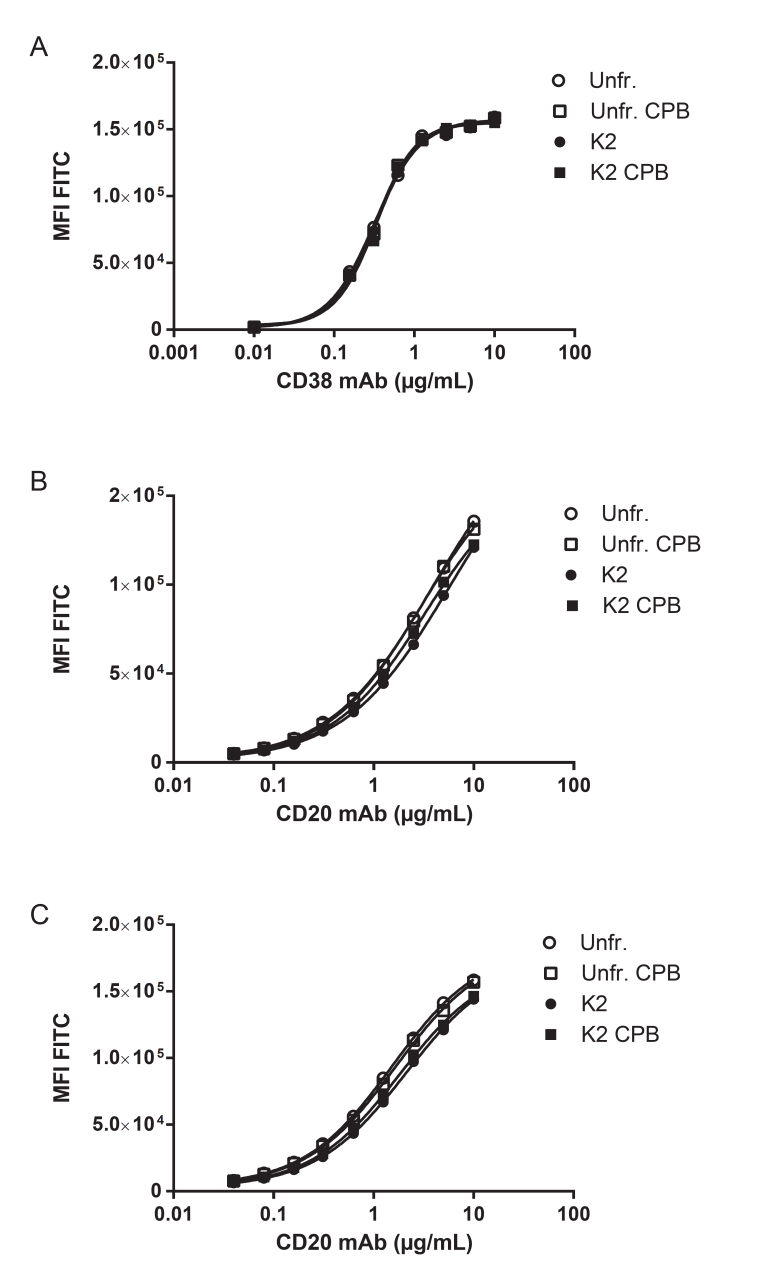


**Supplementary Figure 2.** Antibody binding was assessed via flow cytometry. Dose-response curves plotting the mean fluorescence intensity (MFI) of the FITC-signal versus the mAb concentration are shown. Binding of the unfractionated CD38 mAb and K2 isoform to Daudi cells before and after CPB treatment is shown in panel (A). The unfractionated CD20 mAb and K2 isoform with and without CPB treatment on Daudi and Raji cells are shown in panels (B) and (C), respectively.


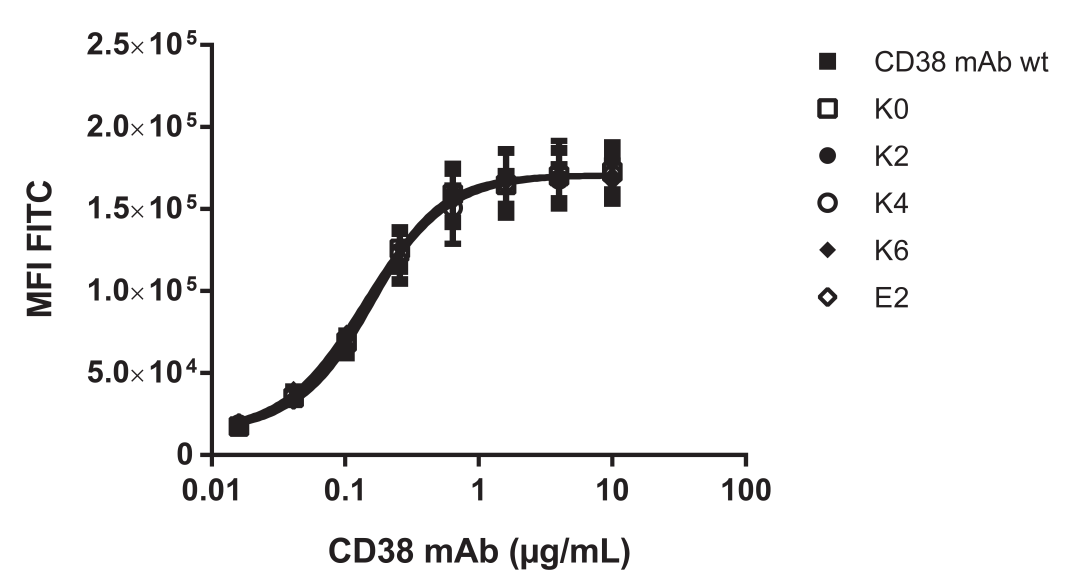


**Supplementary Figure 3.** Binding of wild-type CD38 mAb 005 and the panel of C-terminal mutants was assessed via flow cytometry (n=3). Dose-response curves expressing the mean fluorescence intensity (MFI) of the FITC-signal versus the mAb concentration were plotted.


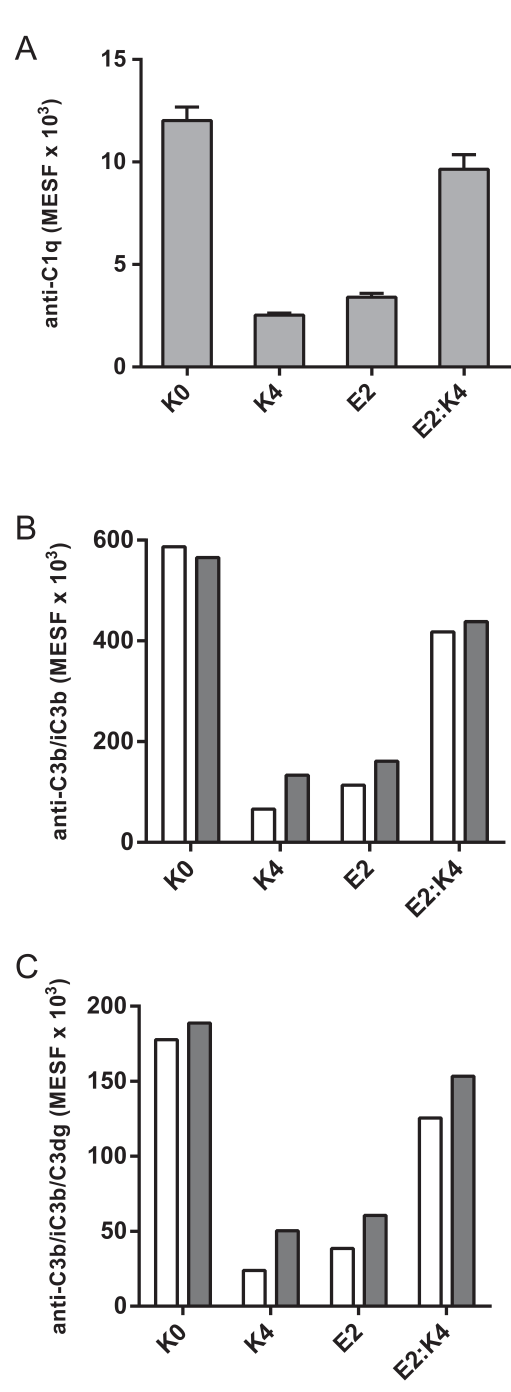


**Supplementary Figure 4.** C1q binding on Daudi cells opsonised with C-terminal mutants. (A) Total mAb concentrations were 20 µg/mL during opsonization and 10 µg/mL during incubation in 50% NHS. The samples were subsequently probed with FITC anti-C1q for 30 min at room temperature (n=2)(B and C) Daudi cells were incubated in 50% NHS supplemented with the indicated mAb or the E2:E4 (1:1) mAb combination at 10 µg/mL (total) for either 2.5 min (open bars) or 30 min (filled bars) at 37ºC. The reaction was stopped by addition of excess cold BSA/PBS. The cells were washed twice and probed with a cocktail of FITC anti-C3b/iC3b mAb 7C12 (B) and Al647 anti-C3b/iC3b/C3dg mAb 1H8 (C)


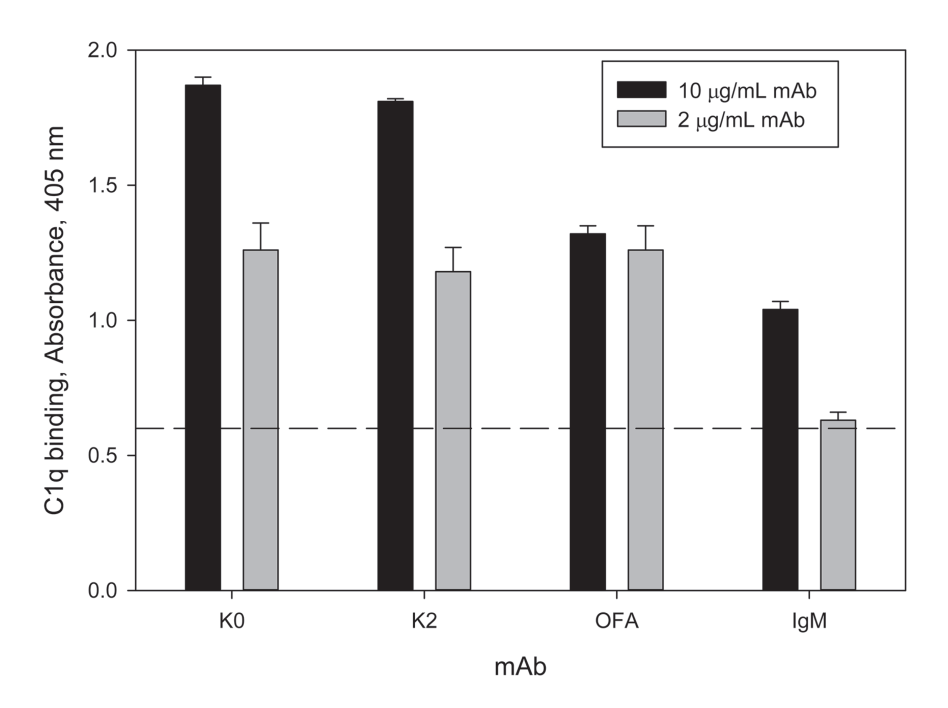


**Supplementary Figure 5.** There is comparable binding of C1q to K0 and to K2 when the mAbs are coated on wells in an ELISA format. OFA and human IgM were used as positive controls. The mAbs were coated at 10 and 2 μg/mL, and then reacted with a 20-fold dilution of NHS, followed by development with rabbit anti-human C1q and then peroxidase conjugated goat anti-rabbit IgG. The horizontal line gives the background signal for samples that were not reacted with NHS.


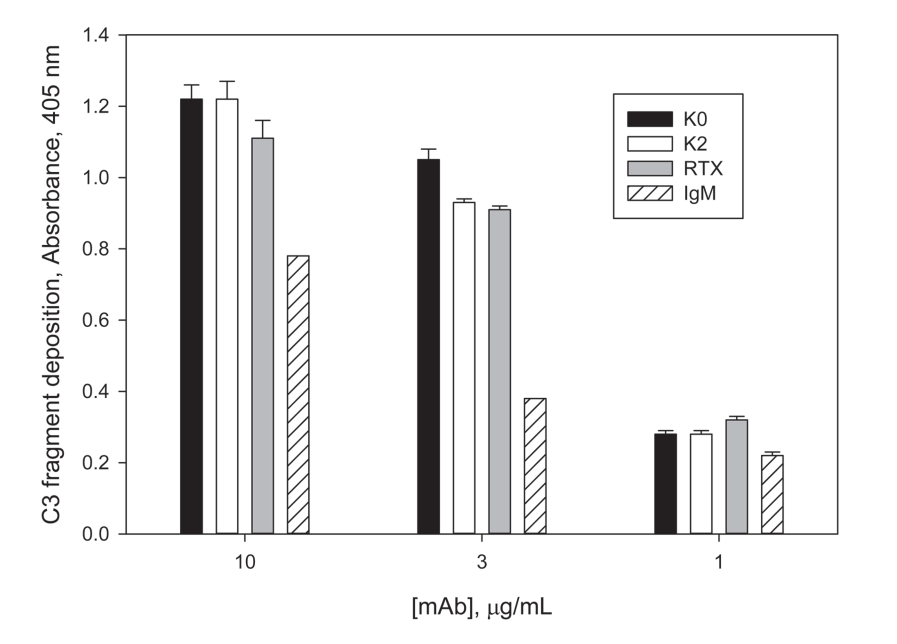


**Supplementary Figure 6.** There is comparable complement-mediated deposition of C3b fragments to K0 and to K2 when the mAbs are coated on wells in an ELISA format. RTX and human IgM were used as positive controls. The mAbs were coated at 10, 3, and 1 μg/mL, and then reacted with neat NHS, followed by development with biotinylated mAb 1H8 (anti-C3b/iC3b/C3d) and then streptavidin peroxidase. The background signal for samples not reacted with NHS was < 0.10.
