# Supplementary material for: Human IgG is produced in a pro-form that requires clipping of C-terminal lysines for maximal complement activation
Source: MAbs. 2015 Jun 2;7(4):672–80. doi: 10.1080/19420862.2015.1046665 (PMC4622059; doi:10.1080/19420862.2015.1046665)
Supplement: Supplemental_Material.zip [file kmab-07-04-1046665-s001.zip › Supplementary Table 1.docx]

**Supplementary Table 1.** Overview of the CD38 C-terminal heavy chain mutants.

| C-terminal sequence | Abbreviation | Side chain charges | pI value |
| --- | --- | --- | --- |
| 445-PG**E**-447 | E2 | - | 8.2 |
| 445-PG-446 | K0 | 0 | 8.5 |
| 445-PG**K**P-448 | K2 | + | 8.8 |
| 445-PG**KK**P-449 | K4 | ++ | 9.0 |
| 445-PG**KKK**P-450 | K6 | +++ | 9.1 |

The C-terminal sequence and accompanying abbreviation is indicated. For each introduced lysine a positive charge (+) and for glutamic acid a negative charge (-) was indicated. The measured pI value of each C-terminal variant was determined by cIEF (**Fig. 4A**).
